# Removal of Chloroxylenol Disinfectant by an Activated Sludge Microbial Community

**DOI:** 10.1264/jsme2.ME18124

**Published:** 2019-02-23

**Authors:** Donggeon Choi, Seungdae Oh

**Affiliations:** 1 Department of Civil Engineering, Kyung Hee University Yongin-si, Gyeonggi-do Republic of Korea

**Keywords:** chloroxylenol, biological wastewater treatment, biodegradation, *Sphingobium*

## Abstract

Chloroxylenol (CHL) is an antimicrobial ingredient that is frequently used in antiseptics/disinfectants for skin (*e.g*. hand soap) and non-living surfaces. CHL is an alternative to triclosan and triclocarban, the use of which has recently been banned in some countries. Accordingly, the more widespread use of CHL may significantly increase its occurrence and level in aquatic environments in the near future, eventually resulting in potential ecological risks. Wastewater treatment plants (WWTPs) may be a point source of CHL in natural environments due to extensive discharge through urban waste stream disposal. While the satisfactory removal of CHL in WWTPs is critical for maintaining healthy aquatic ecosystems, the extent of CHL removal and whether CHL causes system upset/failure in WWTPs currently remain unknown. In the present study, we conducted bioreactor operation and batch experiments to investigate the fate and effects of CHL and elucidate the mechanisms underlying degradation at various levels from environmentally relevant to high levels (0.5–5 mg L^−1^). Bioreactors partially removed CHL (44–87%) via a largely biological route. Microbial association networks constructed using 16S rRNA gene sequencing data revealed selective enrichment and a correlation between *Sphingobium* and CHL, implying its involvement in the biological breakdown of CHL through dehalogenation and ring hydroxylation pathways. The present results provide insights into the behavior and effects of CHL in activated sludge communities and important information for the sustainable management of CHL that may be an emerging issue in the urban water cycle.

Chloroxylenol (CHL) is an active ingredient of various over-the-counter-products, such as skin disinfecting agents, and solutions used to clean surgical instruments in domestic and hospital environments. Since 1920, CHL has been commercially available in a number of formulations, including the handwashing product Dettol (approximately 5% of CHL in the total mixture). CHL exhibits broad spectrum antimicrobial activity and is effective against bacteria, fungi, algae, and viruses. While the exact mechanism of action of CHL currently remains unclear, it includes interactions between the hydroxyl –OH groups of CHL and cytoplasmic membrane proteins in the cell membrane, leading to cell disruption and death (20). CHL may disrupt the human blood cellular composition with repeated oral exposure (11). CHL poses various levels of toxicological risks to freshwater fish (susceptible) and aquatic invertebrates (moderate level) (3). It has also been shown to exert toxicological effects against zebrafish embryos, as demonstrated by its impact on hatching, embryonic mortality, morphology (body curvature), and neurotoxicity (26). A recent study reported the chronic effects of CHL on freshwater fish, which involved genotoxicity and histopathology (3). Previous findings on the toxicological impact of CHL on various organisms from low (*e.g*. bacterial and algal primary producers) to high trophic levels (*e.g*. fish and invertebrates) in aquatic environments strongly suggest its potential to cause environmental health risks.

Despite the widespread use of CHL and its long history, limited data are currently available on its occurrence and behavior in natural and built environments. Previous studies reported that CHL generally occurs at ng L^−1^ to μg L^−1^ levels in aquatic environments, *e.g*. 0.03–0.36 μg L^−1^ (15) and 0.06–1.20 μg L^−1^ (6) in surface river waters. Due to its extensive use in domestic, hospital, and industrial settings, the majority of CHL is carried via sewage disposal to urban wastewater treatment plants (WWTPs), at which it is detected at relatively higher levels up to 65 μg L^−1^ (16). Accordingly, WWTPs represent a point source of CHL discharge into natural environments, which was evident in a previous study (16) showing a markedly higher detection frequency and concentration of CHL in river waters downstream from a WWTP than those upstream of the WWTP. An important finding was the environmental level (>500 μg L^−1^) of CHL detectable in industrially impacted estuarine areas (28). This level significantly exceeded the acute and chronic toxicity thresholds to many aquatic organisms. Since 2016, the Food and Drug Administration in the US has banned the use of the widely used antimicrobials, triclosan and triclocarban, in personal care products based on accumulated evidence of health risks. Consequently, manufacturing communities intend to replace banned products with alternative chemicals, including CHL (26). Accordingly, the more frequent use of CHL in daily life may increase its occurrence in the environment as well as its resulting ecological risks to aquatic ecosystems. Therefore, CHL is a growing concern, and an assessment of its fate and toxicological consequences in the environment that may result from increased use is needed.

WWTPs play a key role in controlling the fate and ecological risk of CHL in the environment because they act as a barrier to limit the spread of various contaminants into downstream bodies of water. With the expected increase in the use of CHL in the near future, toxicological studies on this compound for environmental and human health have begun to emerge (3, 26). Based on environmental management/engineering, it is necessary to establish whether conventional WWTPs (*e.g*. activated sludge) may satisfactorily treat this compound. Therefore, a more complete understanding of the extent of treatment and the mechanisms underlying the degradation of CHL is needed. To date, only one study is available on the removal of CHL (32), and a systematic understanding of the behavior of CHL and its disruptive consequences in activated sludge is currently lacking. The present study aims to provide comprehensive insights into these issues. In contrast to a previous study (32) that examined CHL removal at lower levels (<50 μg L^−1^), we attempted to clarify the behavior of CHL at various levels, including those from environmentally relevant to high levels (500–5,000 μg L^−1^). Our systematic assessment quantified the fate of CHL as well as the mechanisms controlling its behavior in activated sludge, wherein a 16S rRNA gene sequencing analysis of microbial communities exposed to various levels of CHL was employed to assess the effects of CHL on the diversity and structure of the microbial community. The results obtained on CHL removal kinetics and characteristics provide a scientific foundation for the development of strategies for the sustainable treatment and management of CHL in the urban water cycle.

## Materials and Methods

### Chemicals and analytical methods

One liter of substrate contained glucose (1.8 g), KH_2_PO_4_ (0.34 g), K_2_HPO_4_ (0.6 g), CaCl_2_ (0.05 g), MgSO_4_·7H_2_O (0.27 g), NH_4_Cl (0.03 g), FeSO_4_·7H_2_O (0.09 g), and 10 mL of a 100× trace mineral solution. The mineral solution (L^−1^) was composed of ZnSO_4_·7H_2_O (0.35 mg), MnSO_4_·H_2_O (0.21 mg), H_3_BO_3_ (2.1 mg), CoCl_2_·2H_2_O (1.4 mg), CuCl_2_·2H_2_O (0.07 mg), NiSO_4_·6H_2_O (0.14 mg), and Na_2_MoO_4_·2H_2_O (0.21 mg). Chloroxylenol (4-chloro-3,5-dimethylphenol, >98%) was purchased from Alfa Aesar, UK. A 50 g L^−1^ stock solution was prepared in methanol and stored at 4°C until used. A HPLC analytical system equipped with a Shim-Pack GIST Phenyl, 5 μm, 4.6×250 mm column (Shimadze Asia Pacific, Japan) and UV-Vis detector was used to measure CHL concentrations. CHL was detected at 281 nm using an injection volume of 50 μL. Acetonitrile (CAS Number: 75-05-08) was purchased from Merck (Darmstadt, Germany). The mobile phase for the HPLC system was prepared with 60% acetonitrile and 40% water in 25 mM NaH_2_PO_4_ (v/v), adjusted to pH 2.5 with phosphoric acid. The mobile phase was pumped at a flow rate of 1.8 mL min^−1^ under isocratic conditions. Volatile suspended solids (VSS) and chemical oxygen demand (COD) were estimated according to the standard method (1).

### Semi-continuous bioreactor operation

Activated sludge was sampled from an aeration basin of the Jurong Water Reclamation Plant located in Jurong, Singapore. The activated sludge collected was centrifuged at 6,000 rpm for 10 min in a 50-mL conical tube and washed twice with 1× phosphate-buffered saline (PBS) solution. A total of 4 g L^−1^ of activated sludge VSS was inoculated into laboratory fed-batch bioreactors. One-liter reactors (mixed culture volume of 600 mL) were used and 200 mL of the mixed cultures was replaced with the same volume of fresh substrate every 3.5 d (*i.e*. one-cycle duration). The reactors were developed at room temperature (25°C) and aerated using air-stone diffusers to provide 3–4 mg L^−1^ of dissolved oxygen. The pH of the mixed liquor suspension was initially adjusted to pH 7 with 1 N NaOH or 1 N HCL and maintained at pH 6.9±0.1 during entire cycles. The reactors were operated until a steady state was achieved as indicated by operational parameters.

### Batch experiments

Batch tests were performed to describe the removal pathways of CHL occurring in semi-continuous activated sludge bioreactors. Four sets of experiments using sterilized flasks with 100 mL working volumes were established: abiotic, inactive, biotic_CHL+Glc_, and biotic_CHL_. Biotic_CHL+Glc_ was established under identical conditions to those of semi-continuous bioreactors, *i.e*. including an active inoculum and substrate with glucose (1.8 g L^−1^) and 5 mg L^−1^ of CHL. The biotic_CHL_ condition included CHL in the substrate as the sole carbon source (with no glucose). The inactive setting included the inoculum pre-autoclaved (*i.e*. no microbial activity intended). Abiotic lacked an inoculum, and was intended to assess the physicochemical losses of CHL. The inoculum was taken from the mixed culture from reactors fed by 5 mg L^−1^ of CHL plus glucose. It was then centrifuged and washed twice with 1×PBS prior to inoculation. Flasks were incubated with moderate shaking (150 rpm) and at room temperature (23–25°C). The inactive inoculum was made by autoclaving at 121°C and 15 psi pressure for 15 min.

### 16S rRNA gene sequencing and analysis

To investigate shifts in the microbial community structure, the mixed culture suspension of bioreactors was sampled from the control reactors on days 0 and 42, and on day 56 from reactors fed by 0.5 mg L^−1^ (CHL_0.5_) and 5 mg L^−1^ (CHL_5_). All samples were taken in triplicate. DNA extraction and sample preparation for 16S rRNA gene sequencing were conducted as described previously (19). Briefly, the collected mixed liquid suspension was washed twice with 1X PBS, and genomic DNA was extracted using a MoBio PowerSoil^®^ DNA isolation kit (MOBIO, Carlsbad, CA, USA). 16S rRNA sequences (V3–V4 region) were PCR-amplified and sequenced using the MiSeq sequencer at Macrogen (Seoul, Republic of Korea). 16S rRNA gene sequence data were analyzed following the MiSeq SOP pipeline (18) as described previously (19). Briefly, raw sequences were filtered using various parameters (maxambig=0, minimum length=200, maximum length of homopolymer=8 and others at default settings). After filtering, chimeric sequences were checked using the command chimera.vsearch and then discarded. The remaining sequences were taxonomically classified using the command classify.seqs. Sequences affiliated with chloroplasts, mitochondria, unknown, archaea, and eukaryotes were excluded, eventually resulting in 31,000 to 56,000 sequences per sample. Sequences were clustered into operational taxonomic units (OTUs) with a 97% nucleotide similarity cut-off using dist.seqs and cluster commands, which resulted in 235±31, 201±14, and 290±99 for the control, CHL_0.5,_ and CHL_5_ communities, respectively. Sequences were rarefied to 31,000 sequences per sample when estimating the number of OTUs and alpha diversity indices. Statistical testing for differential features (*e.g*. alpha diversity index, community composition, and phenotype) across bioreactors/communities was performed using the Mann-Whitney U test.

### Nucleotide sequence accession number

The 16S rRNA gene sequence datasets used in the present study were deposited in GenBank under the following accession numbers: Control_0_1_ (SRS2340183), Control_0_2_ (SRS2340176), Control_0_3_ (SRS2340220), Control_42_1_ (SRS2340175), Control_42_2_ (SRS2340198), Control_42_3_ (SRS2340197), CHL_0.5_1_ (SRS2340265), CHL_0.5_2_ (SRS2340225), CHL_0.5_3_ (SRS2340226), CHL_5_1_ (SRS2340227), CHL_5_2_ (SRS2340228), CHL_5_3_ (SRS2340229).

## Results and Discussion

### Removal of CHL in bioreactors over time

**Fig. 1:**
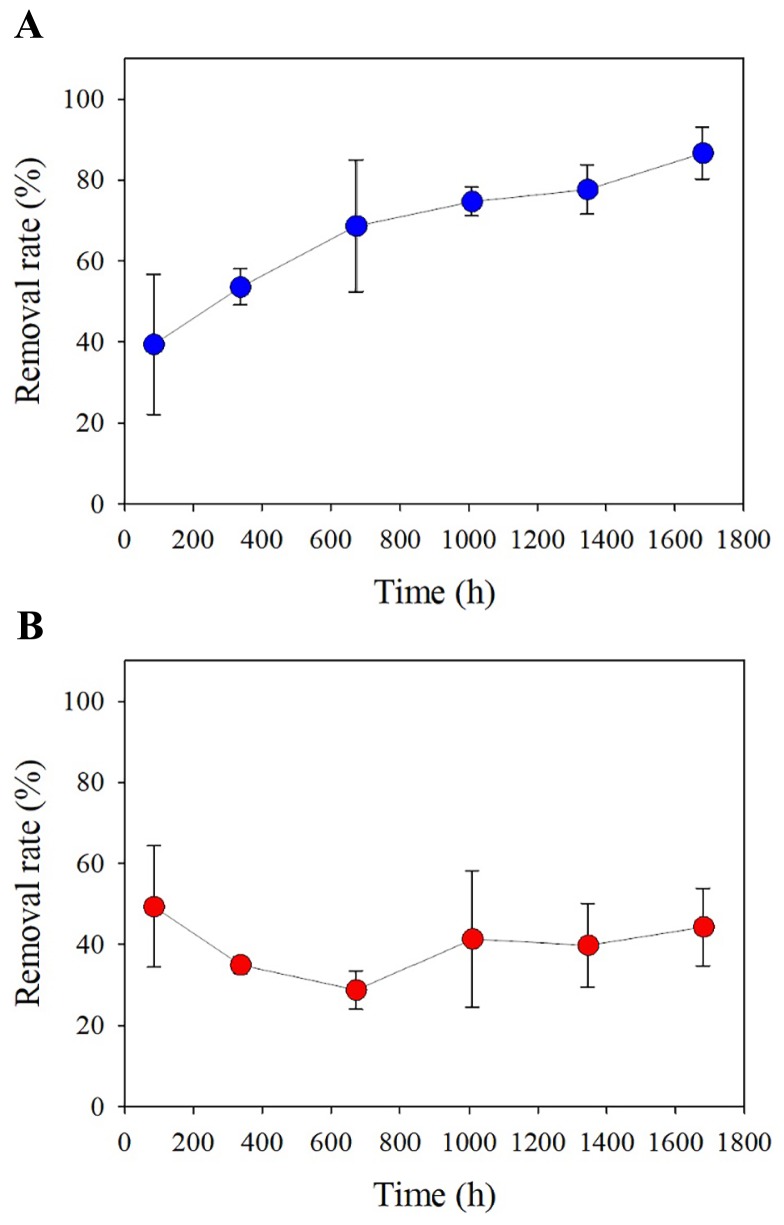
Time course of the removal of CHL in semi-continuous bioreactors. Removal rates in the CHL_0.5_ (A) and CHL_5_ (B) reactors. Error bars indicate the standard deviation from the mean.

Three reactors were fed glucose-containing substrate and acclimated to the laboratory conditions (details described in Materials and Methods) for one month, after which they showed constant COD removal (95%) and MLVSS (0.4 g L^−1^) concentrations, implying quasi-steady state operation. Operational conditions at the steady state were an organic loading rate of 0.4 kg COD kg^−1^ VSS^−1^ d^−1^ and a food-to-microorganism ratio of 0.2 kg COD m^−1^ d^−1^ under a solid retention time of 10.5 d. Two sets of triplicate bioreactors were further developed using the mixed culture suspension of glucose-fed (control) reactors as an inoculum. The two sets were fed by glucose plus 0.5 mg L^−1^ of CHL (CHL_0.5_) and 5 mg L^−1^ of CHL plus glucose (CHL_5_), respectively, and these were maintained separately from the control reactors. Fig. 1 shows the removal of CHL in CHL_0.5_ and CHL_5_ over time. CHL removal in CHL_0.5_ was 39.4±17.3% after 3 d and gradually increased to 53.6±4.4 on day 14 and 68.7±16.3 on day 28. CHL removal in CHL_0.5_ after one month of operation remained at 13.3–25.3% on days 42–70. On the other hand, CHL removal in CHL_5_ at 3 d was 49.4±15.0% and remained relatively stable (39.8–44.3%) over two months.

Some data are available on the occurrence and removal rate of CHL in WWTPs. A 5-month monitoring program reported a 100% detection frequency of CHL in the influents and effluents of wastewaters in two urban WWTPs in the United Kingdom. Specifically, 4–33 and 7–65 μg L^−1^ of CHL in wastewater influents were removed to more than 90% in the two WWTPs employing a trickling filter and activated sludge process as a biological treatment unit, respectively (16); however, the routes of removal remain unclear. An examination of CHL levels in a WWTP in the US showed averages of 0.4 and 0.08 μg L^−1^ of CHL in influent and effluent wastewaters, respectively (32). Our results revealed the more than 80% removal of 500 μg L^−1^ CHL at the steady state, which was consistent with previous estimates in full-scale WWTPs (16, 32). Based on removal efficiencies in WWTPs, contaminants may be classified into those that are poorly (<50%), moderately (50–80%), and highly (>80%) removable (22). CHL at the ~ μg L^−1^ level is considered to be highly removable; however, our results suggested that the input CHL level is a critical factor affecting the removal behavior of CHL in activated sludge. CHL removal at higher levels (5 mg L^−1^) was relatively lower (<50%) over two months, making it very different from that at the 500 μg L^−1^ level. The higher level (5 mg L^−1^) tested in the present study may be entirely relevant for levels associated with high peaks among temporal/spatial variations or accidental discharges when considering the increasing use of CHL in recent years. Furthermore, CHL occurred at 581 μg L^−1^ in industrially impacted estuarine waters (*e.g*. areas around the point discharges of sewage) (28), implying that mg L^−1^ levels of CHL are detectable in municipal waste streams associated with industrial, pharmaceutical, and hospital inputs.

### Co-occurrence network of bacterial taxa under CHL exposure

**Fig. 2:**
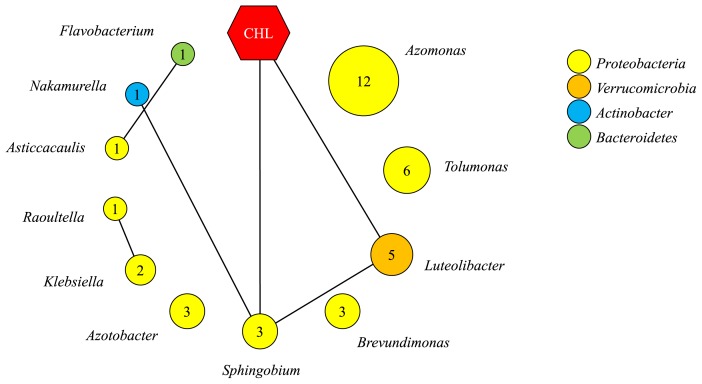
Association networks of bacterial genera. A network analysis of major genera (>1% of relative abundance) was performed using CoNet (8) as described previously (21). The relationship in terms of relative abundance between any two genera was defined based on Pearson’s correlation metric. Each circle represents a genus. The size of the circle represents the average relative abundance of the genus across all communities, and the value is shown in the circle. The circle is color-coded (see key) based on the phylogenetic affiliation. Each edge represents a positive association with >0.6 Pearson’s correlation coefficient and *P*<0.05. No negative association with Pearson’s R<−0.6 with *P*<0.05 was observed.

Long-term exposure to CHL has been speculated to exert selective pressure on activated sludge communities. We hypothesized that there is a discoverable community compositional difference between CHL-exposed and control communities. Fig. S1 shows the relative abundance of major phyla in each sample. *Proteobacteria* (62.4% on average) were the most dominant phylum, followed by *Bacteroidetes* (18.1%), *Verrucomicrobia* (8.3%), and *Actinobacteria* (3.4%). While the abundance of *Proteobacteria* decreased, *Verrucomicrobia* were highly selected (>8-fold change) upon 5 mg L^−1^ of CHL. Fig. S2 shows the list of major genera with 1% or higher relative abundance across communities. Microbial association networks were constructed to examine relationships between the abundance of two bacterial taxa using Pearson’s correlation metric (Fig. 2). A positive correlation (Pearson’s R>0.6 with *P*<0.05) was observed within the three groups: i) *Luteolibacter*, *Sphingobium*, and *Nakamurella*, ii) *Flavobacterium* and *Asticcacaulis*, and iii) *Raoultella* and *Klebsiella*. The relationship between the abundance of each taxon and the exposure level of CHL was also examined, revealing positive correlations with *Luteolibacter* and *Sphingobium*, respectively. The relative abundance of *Luteolibacter* was 16.0±10.7% in CHL_5_, significantly higher than those (0.9±0.3) in the control. *Sphingobium* was also overrepresented in CHL_0.5_ (3.9±1.9%) and CHL_5_ (5.3±2.3%) relative to the control (1.8±1.7%).

*Sphingobium* and *Luteolibacter* are Gram-negative aerobic bacteria that are often found in activated sludge; however, their exact roles in activated sludge ecosystems remain unclear (12, 23). *Sphingobium* are capable of metabolizing a number of contaminants including aromatic, polycyclic aromatic hydrocarbons, chloroaromatic compounds, and pesticides (9, 12, 27). CHL (C_8_H_9_ClO) is a chlorinated phenolic compound that often persists in the environment under aerobic conditions. While dioxygenase is involved in catalyzing the cleavage of aromatic rings, the dioxygenase-mediated reaction may interfere with the chlorine atoms of chlorinated aromatic compounds due to steric and electronic interactions (7). Accordingly, the degradation of chlorinated phenolic compounds is largely due to bacteria capable of catalyzing the removal of chlorine atoms by dehalogenase enzymes, followed by the breakdown of aromatic compounds via dioxygenase-mediated pathways. While no data is currently available on what and how microorganisms degrade CHL, it is important to note that *Sphingobium* carry genes encoding dehalogenase and dioxygenase, the enzymatic activities of which have been experimentally validated (2, 13). Furthermore, *Sphingobium* have demonstrated catabolic activities on monochlorinated dibenzofurans and pentachlorophenol (C_6_HCl_5_O), which are chlorinated and aromatic compounds (2, 13, 31). While *Luteolibacter* are known to inhabit a number of environments including freshwater, activated sludge, seawater, Arctic tundra soil, and the leek rhizosphere (5, 17, 23, 33, 34), the metabolic capacity of *Luteolibacter* remains largely unknown. *Luteolibacter* may also enable the oxygenase-mediated degradation of polycyclic aromatic hydrocarbon compounds (4). Overall, the selective enrichment of *Sphingobium* and *Luteolibacter* systematically observed in our 6 replicate reactors exposed to CHL and the genetic and phenotypic catabolic capacities previously described collectively suggest that the selected bacteria contribute to the removal of CHL measured in the reactor; however, this requires further experimental confirmation. Since *Sphingobium* have shown metabolic versatility for various xenobiotic compounds, such as pyrethroids, toluene, pyrene, and (chlorinated) furans (12, 27, 31), further studies involving the genomic and phenotypic characterization of the microbial agent will contribute to assessments of microbial control over the fate of many micropollutants as well as CHL in urban waste streams.

### Mechanism controlling the fate of CHL

**Fig. 3:**
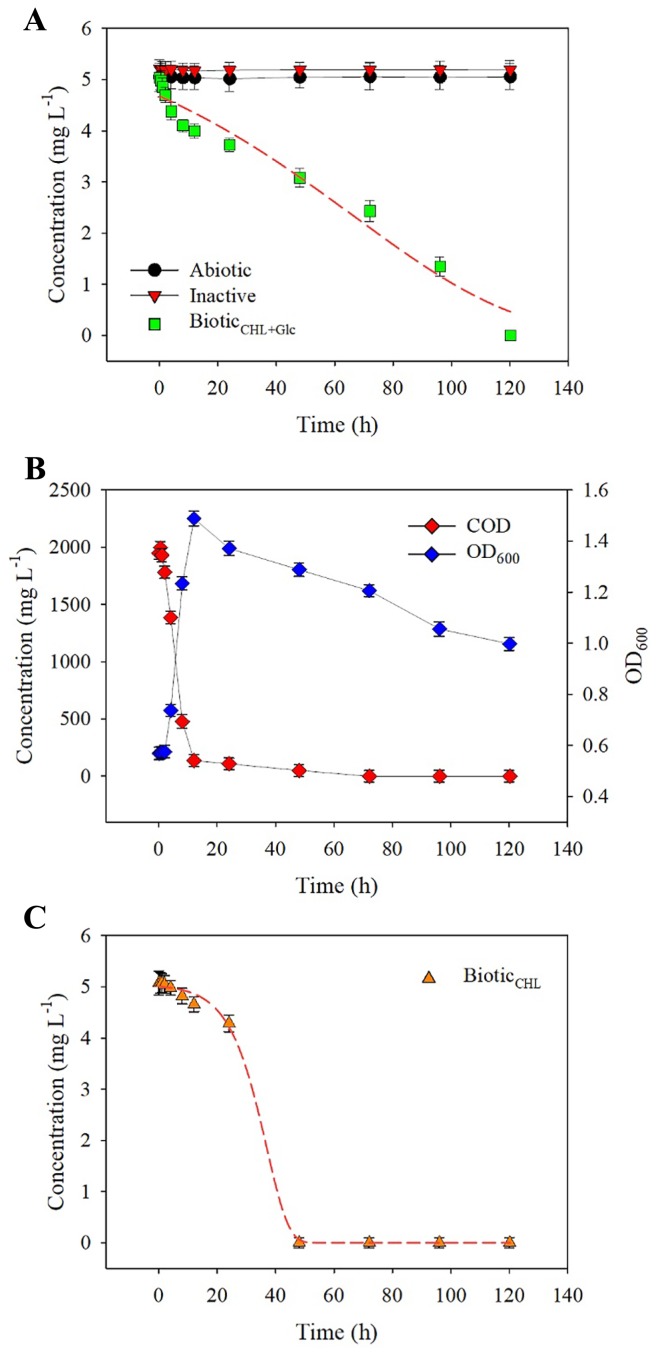
CHL concentration profile in batch experiments. (A) Time course of the removal of CHL in abiotic (●), inactive (○), and biotic_CHL+Glc_ (Δ). (B) COD concentration and cell growth (OD_600_) in the biotic_CHL+Glc_ setting. (C) Time course of the removal of CHL in the biotic_CHL_ setting, in which CHL was used as a sole carbon source. The Modified Gompertz model (red dashed line) was used to fit CHL removal data over time. Error bars indicate the standard deviation from the mean.

The positive correlation observed between *Sphingobium* and the level of CHL exposure (Fig. 2) implied that a certain fraction of CHL removal observed in the bioreactors (Fig. 1) is associated with biological losses. Contaminant removal routes in activated sludge processes generally include, but are not limited to, biodegradation, adsorption (to cells and/or particles), and other abiotic means (*e.g*. volatilization, photolysis, and hydrolysis). We quantitatively assessed the presumable routes of CHL removal occurring in bioreactors. Fig. 3A shows CHL concentration profiles under the three experimental settings. CHL concentration profiles in the abiotic and inactive conditions remained unchanged for 120 h. The mixed culture suspension inoculated into the inactive condition was completely sterilized in advance using an autoclave. The abiotic condition was performed using the same conditions as those in the semi-continuous bioreactors, except for the biomass inoculation. Accordingly, the results obtained demonstrated that CHL was not removed by biosorption (*i.e*. adsorption to cells) or other abiotic routes (*e.g*. volatilization and other physicochemical breakdown). The two biotic conditions (biotic_CHL+Glc_ and biotic_CHL_) showed a decrease in CHL over 120 h (Fig. 3A and C). The majority of cell growth (measured by OD_600_) and heterotrophic activities (measured by COD removal) in the biotic_CHL+Glc_ condition occurred intensively within 24 h (Fig. 3B). Since the mixed culture completely removed CHL under the biotic_CHL_ condition (fed as a sole carbon and energy source) within 48 h, CHL was primarily removed by a biodegradation pathway, in particular, via a direct metabolic, not co-metabolic pathway.

The fate of a micropollutant in biological engineered systems (such as activated sludge processes) is subject to physicochemical characteristics (*e.g*. volatility and sorption affinity) and biodegradability. Henry’s law constant of CHL is 5.1×10^−7^ atm-cu m mole^−1^, suggesting that the volatilization potential of CHL in stripping/aeration in WWTPs is low[Toxicology Data Network. Chloroxylenol. http://toxnet.nlm.nih.gov/cgi-bin/sis/search2/r?dbs+hsdb:@term+@DOCNO+7427 (accessed 6 Aug 2018)]. Physicochemical properties (3.1 of organic carbon-water partition coefficient [logKoc] and 3.3 octanol-water partition coefficient [logKow]) [Toxicology Data Network. Chloroxylenol. http://toxnet.nlm.nih.gov/cgi-bin/sis/search2/r?dbs+hsdb:@term+@DOCNO+7427 (accessed 6 Aug 2018)] suggest a moderate level of hydrophobicity, implying slow mobility in sediment/soil environments. Furthermore, the hydrolysis of CHL in environmental conditions is less likely to be due to the lack of functional groups that undergo hydrolysis. The bioconcentration factor (BCF) indicates the extent of a chemical to partition from water to cellular tissues and is often used as a surrogate for bioaccumulation. The estimated log BCF (1.6) of CHL suggested less biosorption to activated sludge because chemicals with <2 of log BCF generally have a lower bioaccumulation potential. Although the inoculum sludge taken from the CHL_5_ reactors was pre-washed using PBS solution twice prior to the batch experiments, sludge surfaces may sorb, to some extent, CHL following its repeated feeding. Accordingly, the remaining CHL already sorbed on the biomass may contribute to reducing adsorption-mediated removal in the abiotic condition (Fig. 3A). A previous study reported that autoclaving not only sterilizes bacterial activity by denaturing cellular enzymes, but also creates new rough cellular surfaces and holes on cell walls (30). The latter characteristics of the autoclaved cells may provide a larger contact area for the additional biosorption of contaminants, such as metals, than those of unautoclaved cells. Nevertheless, negligible sorption was observed under the inactivated condition (*i.e*. autoclaved dead cells with larger/unsaturated surface areas for potential CHL sorption), which strongly suggested that CHL did not partition to activated sludge cells from the liquid phase in the semi-continuous bioreactors at the steady state.

### Biodegradation characteristics and kinetics of CHL: implications for the management of CHL in urban waste streams

Table 1 shows CHL degradation kinetics and the characteristics of CHL_5_ communities that were illustrated by fitting the data shown in Fig. 3A and C, respectively. The Modified Gompertz model showed a high coefficient of determination (*R*^2^>0.97); therefore, it was used to describe the degradation profile of CHL when supplemented as a sole (Fig. 3C) or dual carbon source (Fig. 3A). The maximum degradation rate (*μ*_m_) and lag phase (*λ*) were 0.05 mg L^−1^ h^−1^ and 3.1 h and 1.13 mg L^−1^ h^−1^ and 23.3 h under the biotic_CHL_ and biotic_CHL+Glc_ conditions, respectively, suggesting strongly differential degradation characteristics depending on the presence of another carbon source other than CHL. When CHL was the sole carbon source, the communities showed a 20-fold higher degradation rate at the expense of a seven-fold longer lag phase.

Toxicological assays reported that CHL caused acute toxicity against invertebrates at 200 μg L^−1^. A recent study demonstrated that CHL has mutagenic consequences (chronic toxicity), even at very low levels (4 μg L^−1^), on the red blood cells of aquatic organisms (rainbow trout) (3). It also disrupts gene expression/regulation and the structure of cell tissues. These findings suggest that CHL poses environmental health risks because detectable levels in wastewater-associated (4–65 μg L^−1^) (16) and industrially impacted (up to 581 μg L^−1^) environments (28) were similar to or exceeded previously demonstrated toxicity levels (4–200 μg L^−1^). Based on the importance of the breakdown of CHL in WWTPs (which are the final barrier limiting the spread of CHL to the environment), satisfactory biodegradation by activated sludge is expected to occur in WWTPs in order to meet safety requirements.

The hydraulic retention time (HRT) is one of the most important factors controlling the fate of a micropollutant. The removal efficiencies of micropollutants often negatively correlate with the half-life time. Compounds with a half-life time less than the HRT of a WWTP generally showed high removal efficiencies (29). The half-life time of CHL was 34–56 h based on the modified Gompertz model using the data of Fig. 3A and C. This half-life significantly exceeded the typical HRTs (4–8 h) in conventional activated sludge processes. Accordingly, our kinetic data on 5 mg L^−1^ of CHL suggested the poor removal of CHL in conventional activated sludge processes. Furthermore, the biodegradation constant (K_biol_, L g^−1^ VSS^−1^ d^−1^) may be used to represent the pollutant biodegradability of a micropollutant, providing information on the potential degree of removal in WWTPs. The classification scheme for a micropollutant based on (14) includes categories for no substantial biodegradation (K_biol_<0.1), partially biodegradable (0.1<K_biol_<10), and readily biodegradable (K_biol_>10). The K_biol_ constant values of CHL were calculated using our experimental data based on Fig. 3A. The K_biol_ constant was 1.8 L g^−1^ VSS^−1^ d^−1^ under the biotic_CHL+Glc_ condition (Fig. 3A). Using the model prediction (14) and K_biol_ constant value measured in the present study, CHL-bearing waste streams appeared to be partially treated by conventional plug flow activated sludge processes with HRT of 12 h and SRT of 10–15 d, resulting in approximately 20% of the residual CHL load in the wastewater effluent. These predictions are consistent with previously measured removal efficiencies in full-scale WWTPs (32).

Based on the levels of CHL (500–5,000 μg L^−1^) and removal rates (up to 80%) in CAS processes that were predicted in our laboratory experiments and experimentally confirmed in previous studies, the expected residual loads in effluents may not meet the stringent safety criteria associated with the toxicity threshold (4–200 μg L^−1^). There is currently no reliable treatment alternative to assure the satisfactory removal of diverse micropollutants. Several advanced oxidation processes (AOPs) with UV/O_3_, sonochemical, and electrochemical methods have been tested for treating CHL-bearing waste streams. The sonochemical treatment catalyzes the dechlorination of CHL, resulting in more than 75% degradation within 24 h (10). The electro-Fenton and UV/O_3_ processes achieve the complete decomposition of CHL in 15 and 6 min, respectively (24, 25). These advanced treatment technologies using AOPs exhibit excellent treatment efficiencies in a short-time scale, and, thus, may be appropriate for the sustainable management of CHL in combination with existing biological unit processes in WWTPs.

## Conclusion

We herein elucidated the mechanism controlling the fate of CHL in activated sludge for 70 d of bioreactor operation. The removal of CHL was attributed to biodegradation without considerable physicochemical losses (*e.g*. sorption and volatilization). The biodegradation characteristics and kinetics of CHL varied significantly depending on the input level: 87% removal at 0.5 mg L^−1^ and 44% at 5 mg L^−1^ of CHL. A microbial co-occurrence network analysis identified a bacterial population, *Sphingobium*, that potentially broke down CHL via dehalogenation and aromatic ring hydroxylation. Biodegradation kinetic data (1.8 L g^−1^ VSS^−1^ d^−1^) suggested that CHL in conventional activated sludge processes in full-scale WWTPs under typical operational parameters is not treated down to satisfactory levels that prevent ecological risks to receiving bodies of water. Collectively, the present results provide comprehensive insights into the fate of CHL with its exact mechanism of action and kinetics as well as effects on activated sludge communities, which will be a useful scientific basis for developing suitable treatments and the management of CHL in the urban water cycle against the increased usage of CHL that is expected in the near future.

## SUPPLEMENTARY MATERIAL



## Figures and Tables

**Table 1:** Kinetic parameters of CHL biodegradation

Modified Gompertz model: $C=C_{0}-\text{A}\times \text{exp}\left\{-\text{exp}\left[\frac{\mu_{m}e}{A}\times \left(\lambda -t\right)+1\right]\right\}$				
Condition	*R*^2^	*A* (mg L^−1^)^a^	*μ*_m_ (mg L^−1^ h^−1^)^b^	*λ* (h)^c^
Biotic_CHL_	0.99	5.10±0.09	1.13±0.01	23.3±0.01
Biotic_CHL+Glc_	0.97	5.46±0.07	0.05±0.01	3.09±0.21

a *A* is the biodegradation potential.

b *μ*_m_ is the maximum biodegradation rate.

c *λ* is the phase lag time.
