# Supplementary material for: Removal of Chloroxylenol Disinfectant by an Activated Sludge Microbial Community
Source: Microbes Environ. 2019 Feb 23;34(2):129–35. doi: 10.1264/jsme2.ME18124 (PMC6594741; doi:10.1264/jsme2.ME18124)
Supplement: Supplementary file 1 [file 34_129_s1.pdf]

SUPPLEMENTARY MATERIAL

Removal of chloroxylenol disinfectant by activated sludge microbial community

Seungdae Oh* and Donggeon Choi

Department of Civil Engineering, Kyung Hee University, Yongin-si, Gyeonggi-do, Republic of Korea

SUPPLEMENTARY FIGURES

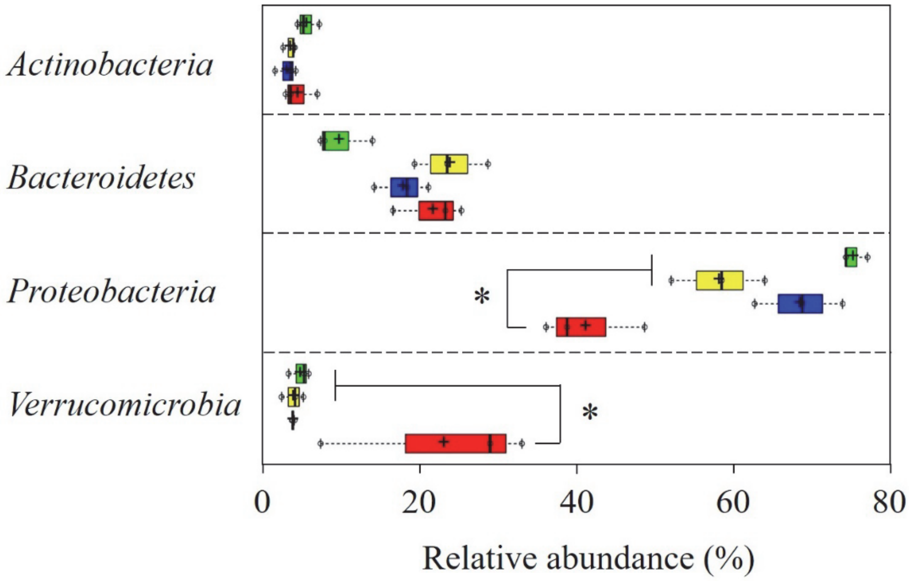

Figure S1. Relative abundance of major phyla in the CHL-exposed and control communities. Boxplot represents the relative abundance (%) of major phyla (> 1% of average) found in the $CHL_{0.5}$ (blue), CHL_5 (red), and control communities (day 0; green, day 42; yellow). Data points are represented by open circles. Whiskers extend to data points that are less than 1.5 times the interquartile range (IQR) away from $1^{st}/3^{rd}$ quartiles; A cross mark represents sample means. An asterisk indicates significantly different abundance in CHL-exposed communities compared to any subgroup of control communities: CHL-exposed vs. those sampled at day 0 [n = 3], CHL-exposed vs. those at day 42 [n = 3], and CHL-exposed vs. the combined [n = 6] of both.

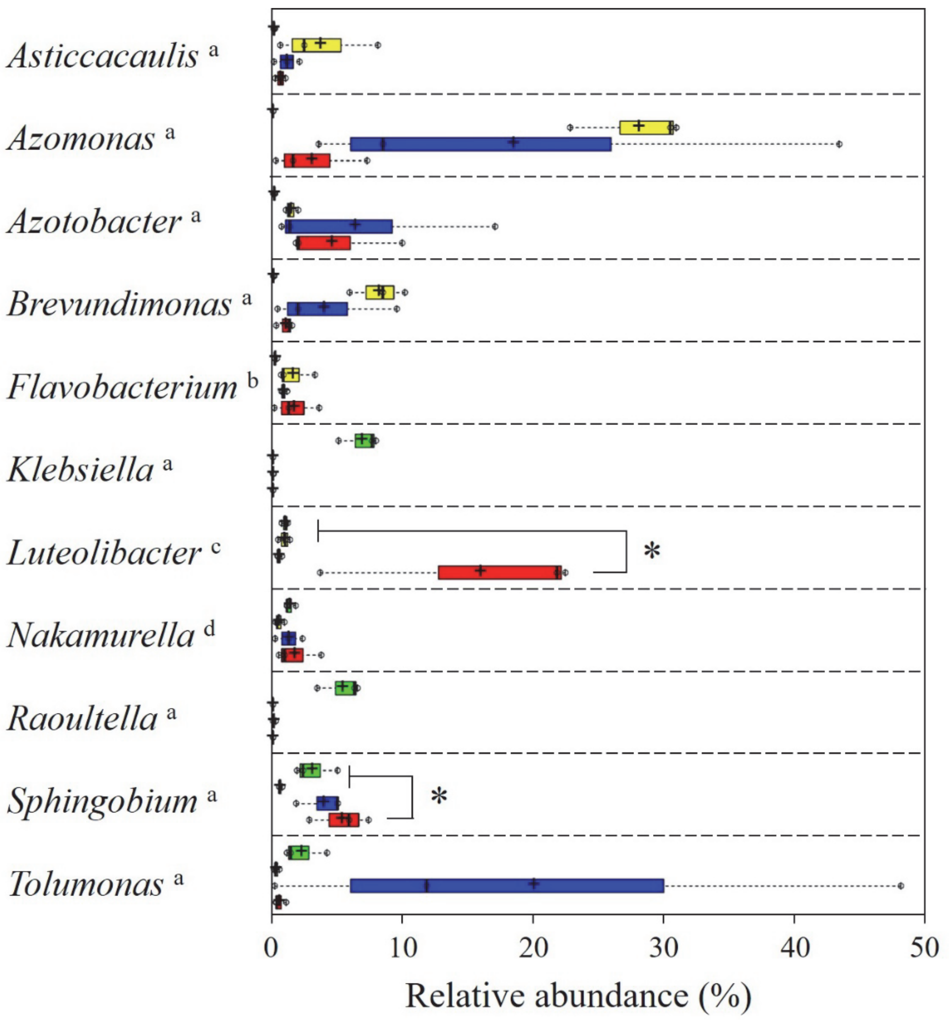

Figure S2. Relative abundance of major genera in the CHL-exposed and control communities. Boxplot represents the relative abundance (%) of major genera (> 1% of average) found in the CHL0.5 (blue), CHL5 (red), and control communities (day 0; green, day 42; yellow). 55% of the sequences could be classified at genus level using the MOTHUR pipeline used in this study. The 11 major genera accounted for 39% of the total classified. An asterisk indicates significant enrichment of taxon in CHL-exposed communities compared the control. Higher classification at the phylum level was shown as denotes; [a] *Proteobacteria*, [b] *Bacterioidetes*, [c] *Verrucomicrobia*, [d] *Actinobacteria*.
